# Supplementary material for: Combining Straight‐Line and Map‐Based Distances to Investigate the Connection Between Proximity to Healthy Foods and Disease
Source: Stat Med. 2025 Apr 14;44(7):e70054. doi: 10.1002/sim.70054 (PMC11995689; doi:10.1002/sim.70054)
Supplement: Supplementary file 1 — Data S1. Supporting Information. [file SIM-44-0-s002.pdf]

Supporting Information for "Combining straight-line and map-based distances to investigate the connection between proximity to healthy foods and disease"

Sarah C. Lotspeich, Ashley E. Mullan,
Lucy D'Agostino McGowan, and Staci Hepler
Department of Statistical Sciences, Wake Forest University

January 19, 2025

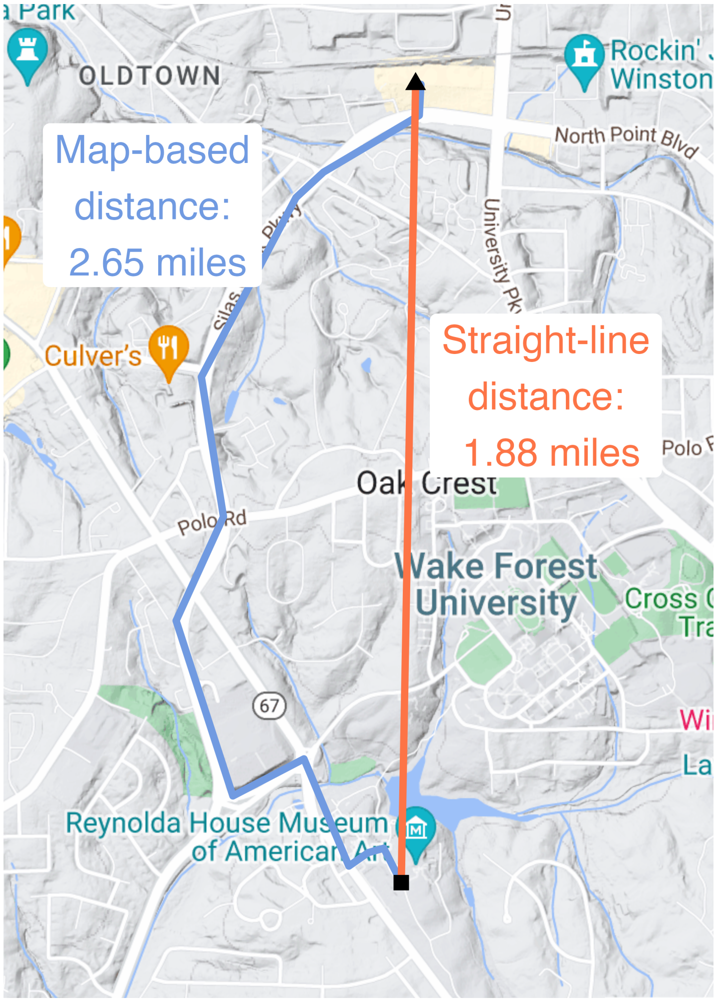

Figure S1: Straight-line and map-based distances from Reynolda House (square symbol) to a nearby Food Lion grocery store (triangle symbol) in Winston-Salem, North Carolina. It can be seen that the estimated driving distance (2.65 miles) was 1.6 times the straight-line distance between them (1.64 miles).

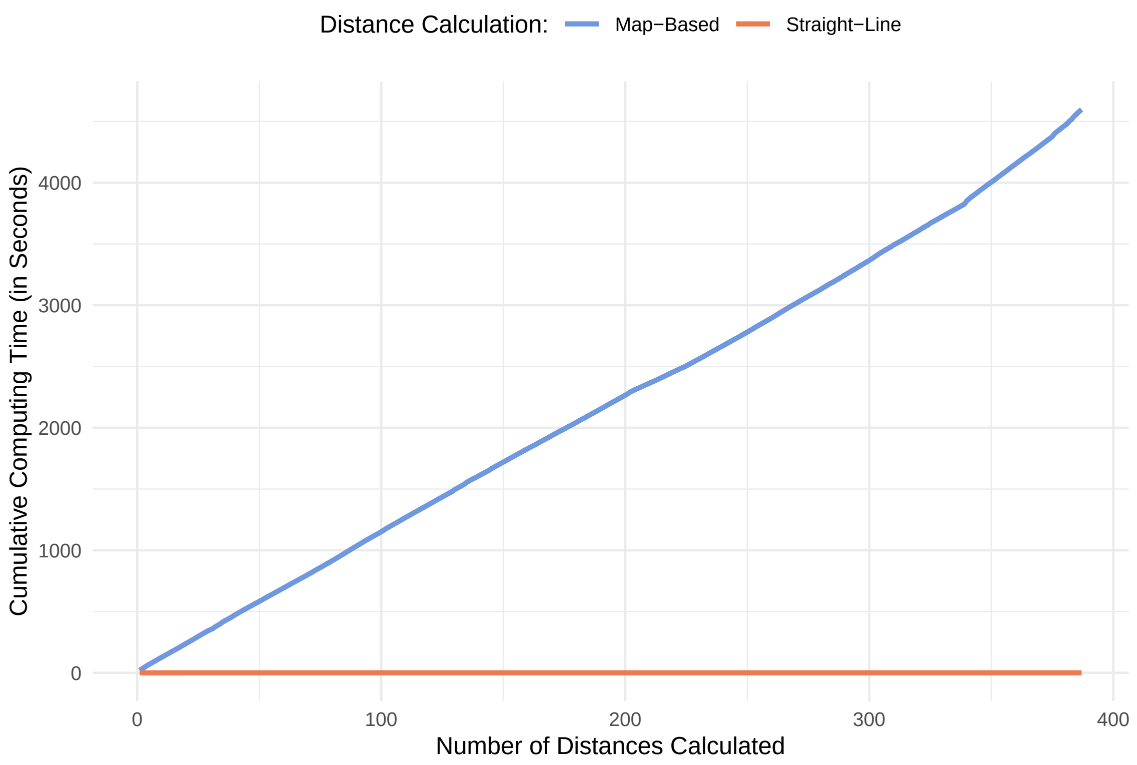

Figure S2: Line graph of the cumulative computing time (in seconds) for the map-based versus straight-line distance calculations in the Piedmont Triad data.

S.1 Including the Outcome in the Imputation Model

As recommended in Moons et al. (2006) and others, the analysis model outcome must be included in the predictor's imputation model for the imputation model to be *congenial*. Since the outcome in a Poisson regression model is transformed:

$$\log\{\mathrm{E}(Y|X)\} = \beta_0 + \beta_1 X + \log(Pop)$$
$$\rightarrow \log\left\{\frac{\mathrm{E}(Y|X)}{Pop}\right\} = \beta_0 + \beta_1 X. \quad \text{(S.1)}$$

Specifically, the analysis model outcome Y is being (i) divided by the offset Pop and then (ii) log-transformed. Thus, there were many possible ways of including Y in the imputation model for X considered.

Recall that the imputation model for map-based access X (described in Section 2.3) is a linear regression with X as the outcome. In a brief simulation study, five possible ways to include the health outcomes from the analysis model in the imputation model were considered:

1. $\mathrm{E}(X|X^*)$, ignoring the outcome and offset,
2. $\mathrm{E}(X|X^*, Y)$, including the untransformed outcome,
3. $\mathrm{E}\{X|X^*, \log(Y)\}$, including the transformed outcome,
4. $\mathrm{E}\{X|X^*, \log(Y/Pop)\}$, including the transformed prevalence, and
5. $\mathrm{E}\{X|X^*, \log(Y), \log(Pop)\}$, including the transformed outcome and offset.

Data were simulated following the data generating mechanism outlined in Section 3.1 for samples of $N = 387$ and 2169 neighborhoods, with a proportion of $q = 0.1$ that had map-based food access X available. An error mean and standard deviation of $\mu_U = -0.7$ and $\sigma_U = 0.8$, respectively, were assumed, and the outcome prevalence and prevalence ratio for X were approximately 11% and 1.01, respectively. Results from these simulations are displayed in Figure S3.

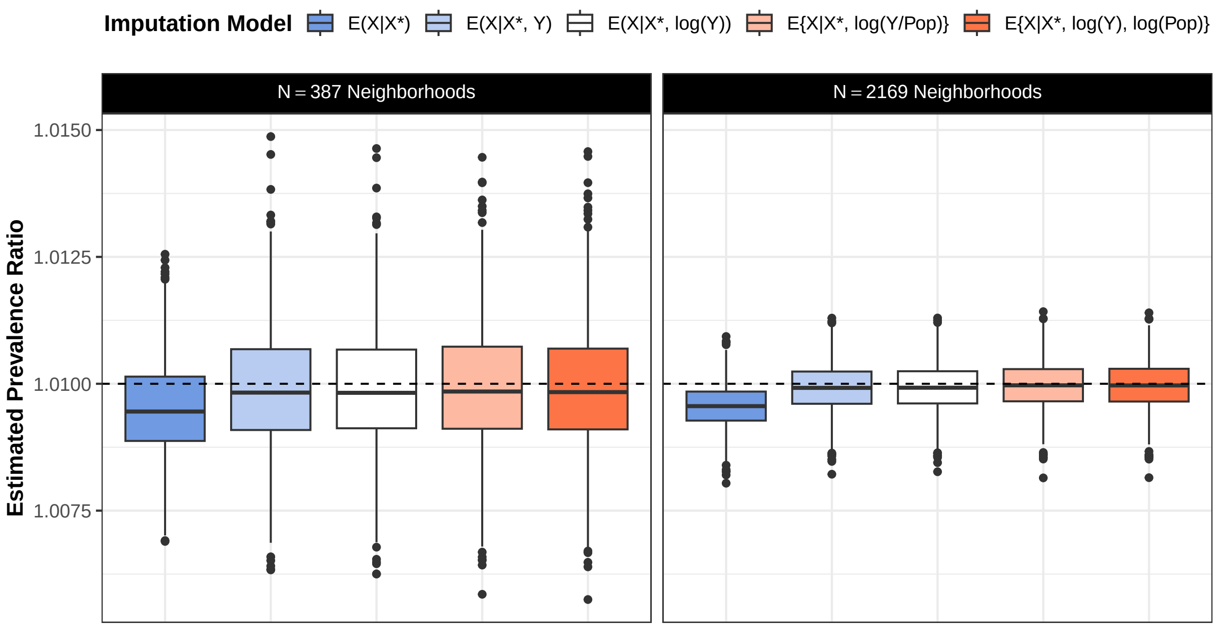

Figure S3: Estimated prevalence ratios for map-based food access X on health using multiple imputation with $B = 20$ imputations. The five possible ways to include the analysis model outcome Y (with or without the model offset Pop) in the imputation model for X were considered. All results are based on 1000 replications.

Imputing X from a model that excluded the outcome entirely led to estimates of the prevalence ratio that were consistently lower than the truth in both sample sizes considered, albeit by a small amount given the small value of β_1. This bias is consistent with the recommendations from Moons et al. (2006) and D'Agostino McGowan et al. (2024). Including the untransformed Y or transformed outcome $\log(Y)$ in the imputation model led to unbiased estimates, on average. Incorporating the offset Pop into the imputation model, either in the log prevalence $\log(Y/Pop)$ or on its own as $\log(Pop)$, could lead to estimates with slightly more variability, but they were still unbiased. Based on these results, missing X values were replaced with random draws from the conditional distribution of X given X^* and $\log(Y)$, corresponding to imputation model 3 considered.

Table S1: Simulation results under varied additive errors in straight-line proximity to healthy foods, as controlled by the mean μ_U of the errors U. The standard deviation $\sigma_U = 0.8$ of the errors was fixed.

		Gold Standard		Naive		Complete Case			Imputation				
N	μ_U	Bias	ESE	Bias	ESE	Bias	ESE	RE	Bias	ESE	ASE	CP	RE
387	−0.10	0.007	0.001	0.032	0.001	0.001	0.003	0.079	0.000	0.001	0.001	0.959	0.713
	−0.35	−0.003	0.001	0.028	0.001	0.009	0.003	0.086	−0.015	0.001	0.001	0.954	0.722
	−0.70	−0.003	0.001	0.043	0.001	−0.004	0.003	0.083	−0.015	0.001	0.001	0.968	0.640
	−1.00	0.000	0.001	0.062	0.001	0.000	0.003	0.086	−0.013	0.001	0.001	0.971	0.625
2169	−0.10	−0.001	0.000	0.024	0.000	−0.005	0.001	0.094	−0.004	0.000	0.001	0.969	0.723
	−0.35	−0.001	0.000	0.031	0.000	−0.003	0.001	0.095	−0.006	0.000	0.001	0.970	0.716
	−0.70	0.001	0.000	0.045	0.000	0.003	0.001	0.100	−0.006	0.000	0.001	0.974	0.658
	−1.00	0.002	0.000	0.063	0.000	−0.002	0.001	0.091	−0.006	0.000	0.001	0.983	0.608

Note: **Bias** and **ESE** are, respectively, the empirical relative bias and standard error of the log prevalence ratio estimator $\hat{\beta}_1$; **ASE** is the average of the standard error estimator $\widehat{\text{SE}}(\hat{\beta}_1)$; **CP** is the empirical coverage probability of the 95% confidence interval for the log prevalence ratio β_1; **RE** is the empirical relative efficiency to the Gold Standard. All entries are based on 1000 replicates.

Table S2: Simulation results under varied multiplicative errors in straight-line proximity to healthy foods, as controlled by the mean μ_W of the errors W. The standard deviation $\sigma_W = 0.15$ of the errors was fixed.

		Gold Standard		Naive		Complete Case			Imputation				
N	μ_W	Bias	ESE	Bias	ESE	Bias	ESE	RE	Bias	ESE	ASE	CP	RE
387	0.30	0.004	0.001	1.301	0.003	0.027	0.003	0.085	−0.038	0.002	0.002	0.935	0.219
	0.50	0.001	0.001	0.700	0.002	0.002	0.003	0.085	−0.031	0.002	0.002	0.912	0.286
	0.70	0.001	0.001	0.335	0.001	0.013	0.003	0.086	−0.007	0.001	0.001	0.937	0.466
2169	0.30	0.000	0.000	1.280	0.001	0.001	0.001	0.086	−0.031	0.001	0.001	0.959	0.201
	0.50	−0.001	0.000	0.691	0.001	−0.004	0.001	0.092	−0.019	0.001	0.001	0.947	0.282
	0.70	0.003	0.000	0.335	0.001	0.006	0.001	0.098	−0.005	0.001	0.001	0.934	0.384

Note: **Bias** and **ESE** are, respectively, the empirical relative bias and standard error of the log prevalence ratio estimator $\hat{\beta}_1$; **ASE** is the average of the standard error estimator $\widehat{\text{SE}}(\hat{\beta}_1)$; **CP** is the empirical coverage probability of the 95% confidence interval for the log prevalence ratio β_1; **RE** is the empirical relative efficiency to the Gold Standard. All entries are based on 1000 replicates.

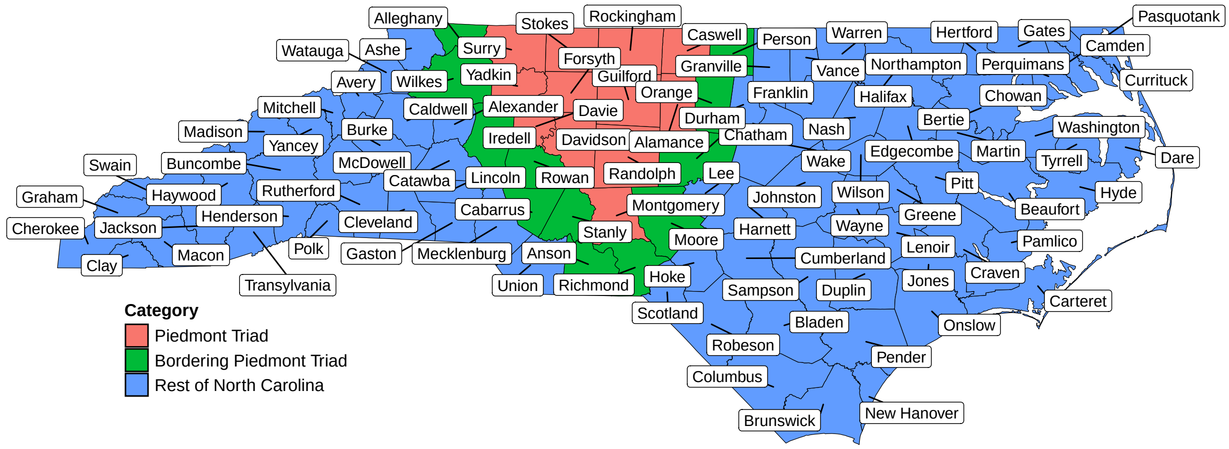

Figure S4: Map of the 100 North Carolina counties, colored by whether they belong to the Piedmont Triad ($n = 12$), border the Piedmont Triad ($n = 12$), or fall into the rest of the state ($n = 76$).

Table S3: Descriptive statistics of the $N = 387$ census tracts in the Piedmont Triad, North Carolina

**Variable**	**Level**	**Summary**
**County**	Alamance	36 (9.3%)
	Caswell	6 (1.6%)
	Davidson	34 (8.8%)
	Davie	7 (1.8%)
	Forsyth	93 (24.0%)
	Guilford	118 (30.5%)
	Montgomery	6 (1.6%)
	Randolph	28 (7.2%)
	Rockingham	21 (5.4%)
	Stokes	9 (2.3%)
	Surry	22 (5.7%)
	Yadkin	7 (1.8%)
**Population**^{a}		4095 (3901 – 5282)
**Percent Population by Race**^{b}	White Alone	0.78 (0.58 – 0.89)
	Black Alone	0.14 (0.05 – 0.32)
	Asian Alone	0.01 (0.00 – 0.03)
	American Indian and Alaska Native Alone	0.00 (0.00 – 0.01)
	Native Hawaiian and Other Pacific Islander Alone	0.00 (0.00 – 0.00)
**Land Area (in Miles**^{2}**)**^{a}		4.70 (1.80 – 19.15)
**Population Density (per Mile**^{2}**)**^{a}		842.90 (230.80 – 2037.45)
**Proximity to Healthy Foods (in Miles)**	Straight-Line	1.01 (0.57 – 2.12)
	Map-Based	1.51 (0.89 – 2.99)
**Prevalence of Health Outcome**^{c}	Diagnosed Diabetes	0.11 (0.09 – 0.13)
	Obesity	0.34 (0.31 – 0.38)

Note: County was summarized by the count (proportion) of census tracts at each level. All other variables were summarized numerically by the median (interquartile range [IQR]). ^{a}Taken from the 2010 U.S. Census. ^{b}Taken from the 2015 American Community Survey. ^{c}Taken from the 2022 PLACES dataset (Centers for Disease Control and Prevention, 2022).

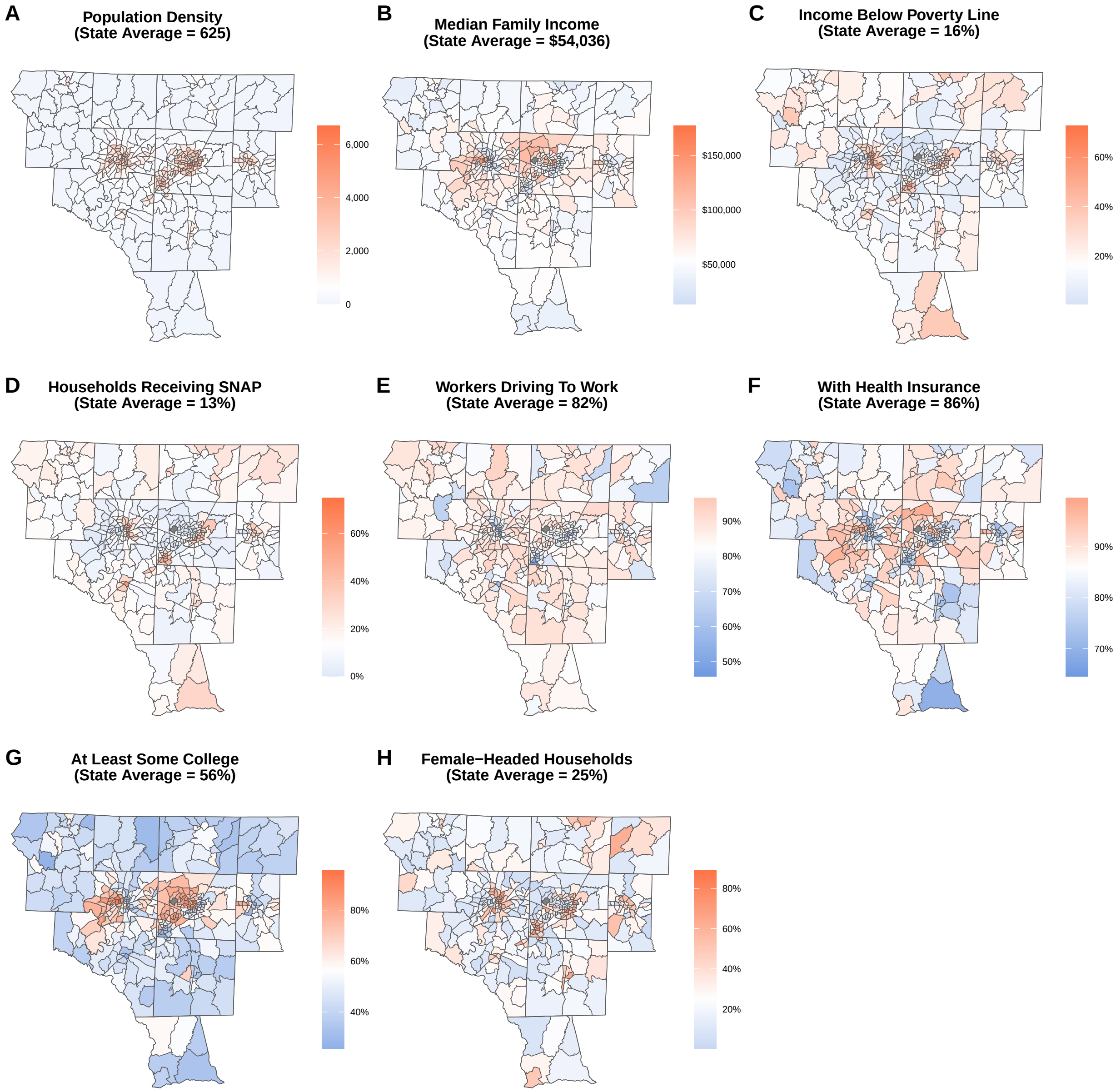

Figure S5: Choropleth maps of socioeconomic factors across the census tracts of the Piedmont Triad, North Carolina. Data were taken from the 2015 American Community Survey. The gradient for each map is centered at the state average (median). Two census tracts were missing median family income. All other maps are based on $N = 387$ census tracts. The one census tract with zero population was excluded from all maps.

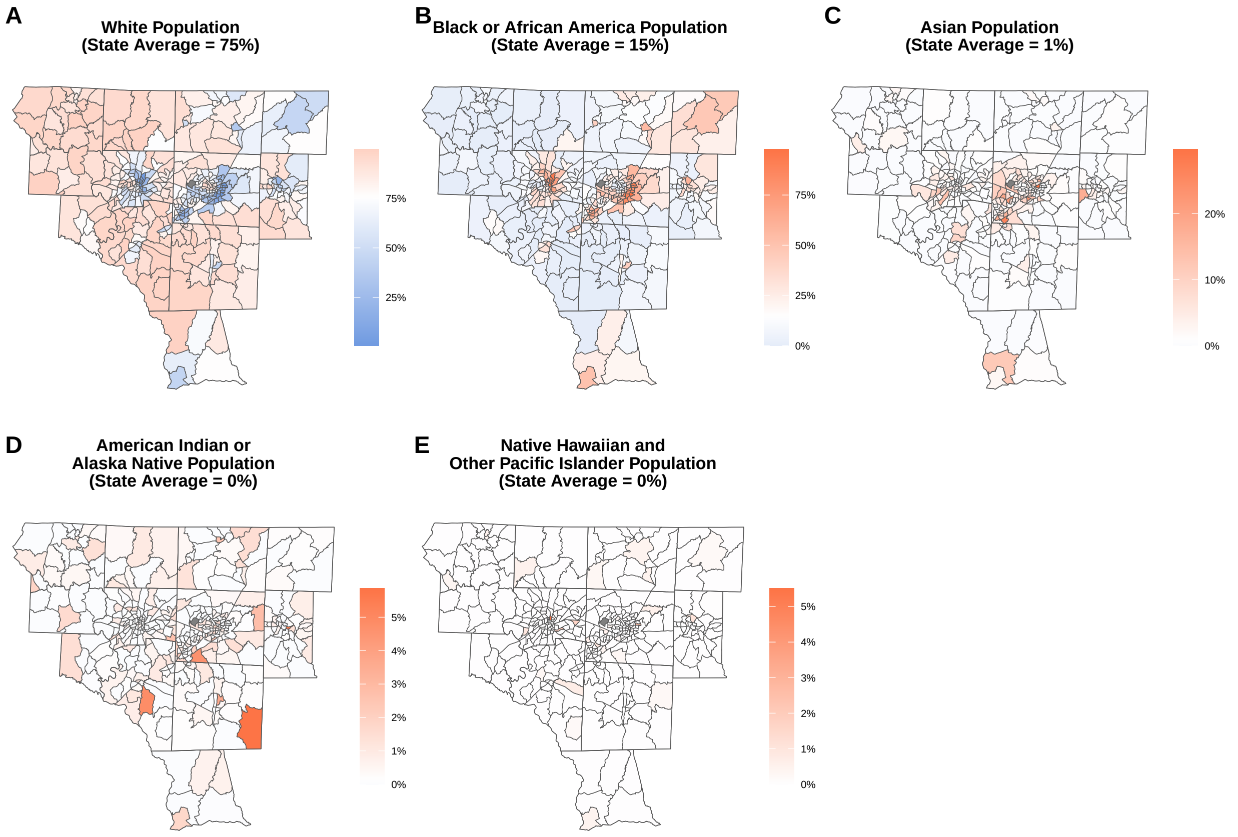

Figure S6: Choropleth maps of percents of census tract population by self-reported race in the Piedmont Triad, North Carolina. Data were taken from the 2015 American Community Survey. The gradient for each map is centered at the state average (median). All maps are based on $N = 387$ census tracts. The one census tract with zero population was excluded from all maps.

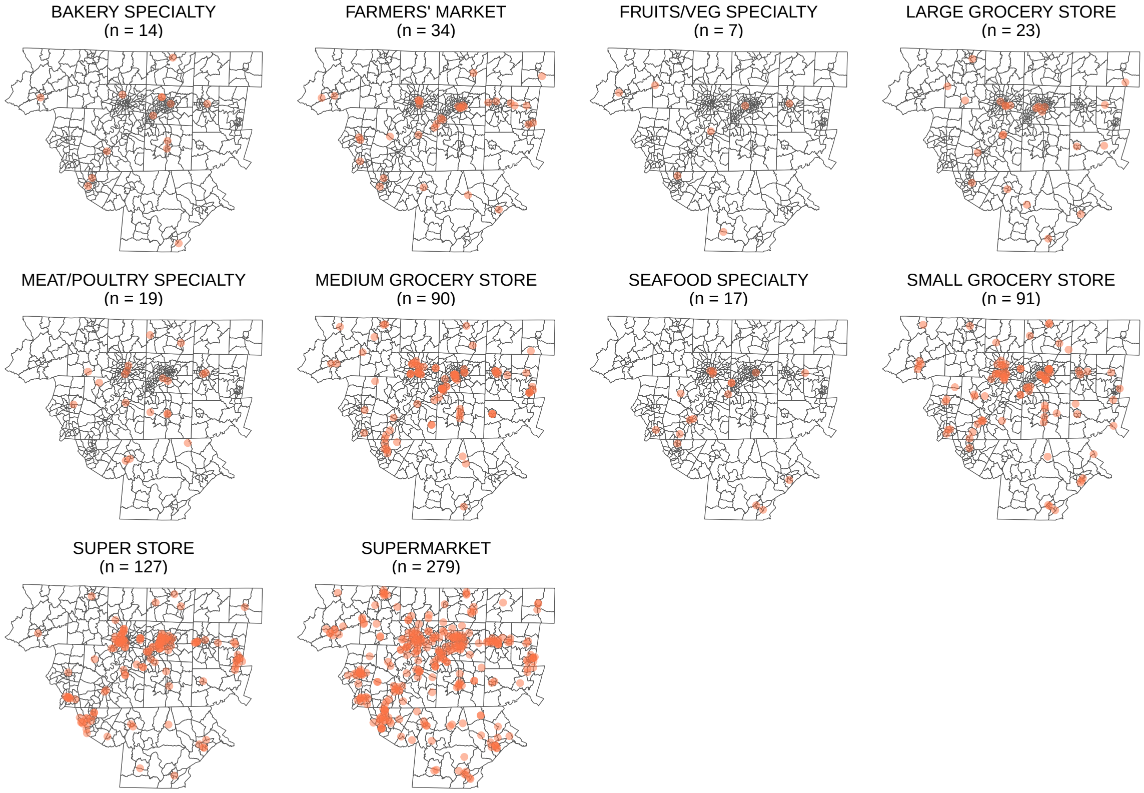

Figure S7: Map of $M = 701$ authorized SNAP retailers in the Piedmont Triad, North Carolina, broken down by store type. Data were taken from the 2022 Historical SNAP Retail Locator Data (United States Department of Agriculture, 2022).

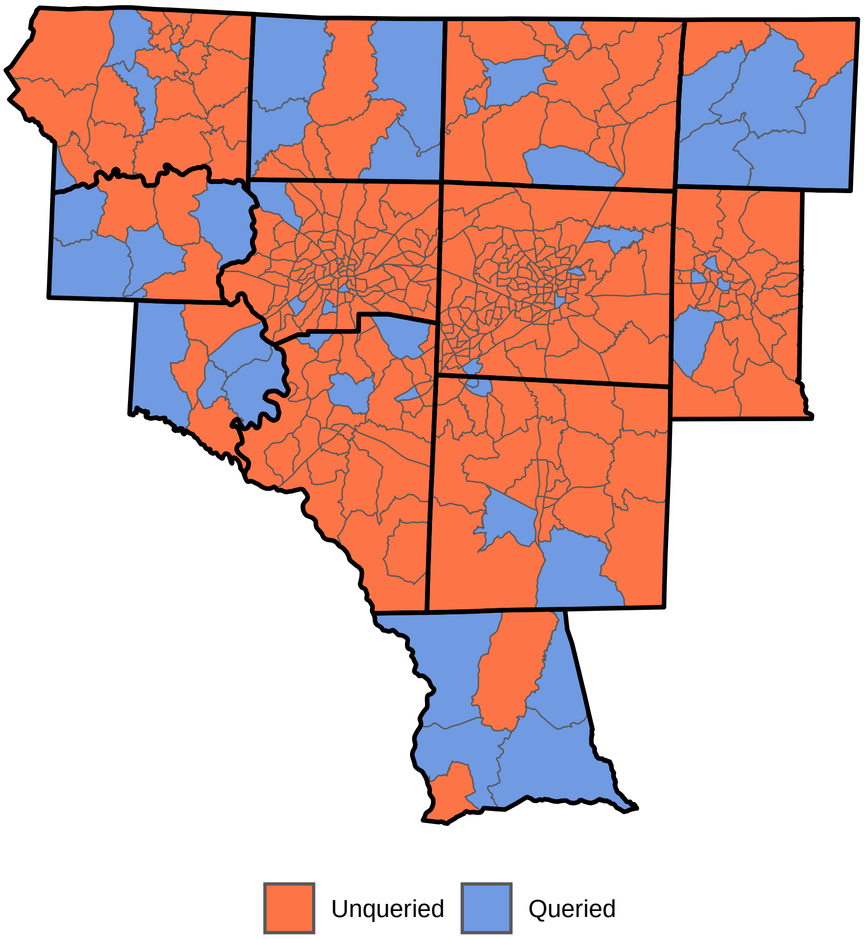

Figure S8: Map of census tracts in the Piedmont Triad, North Carolina, colored according to whether it was treated as queried in the partially queried analysis. For the partially queried analysis, $n = 48$ tracts were chosen to be queried via county-stratified random sampling. The thicker boundaries denote outline the twelve counties.

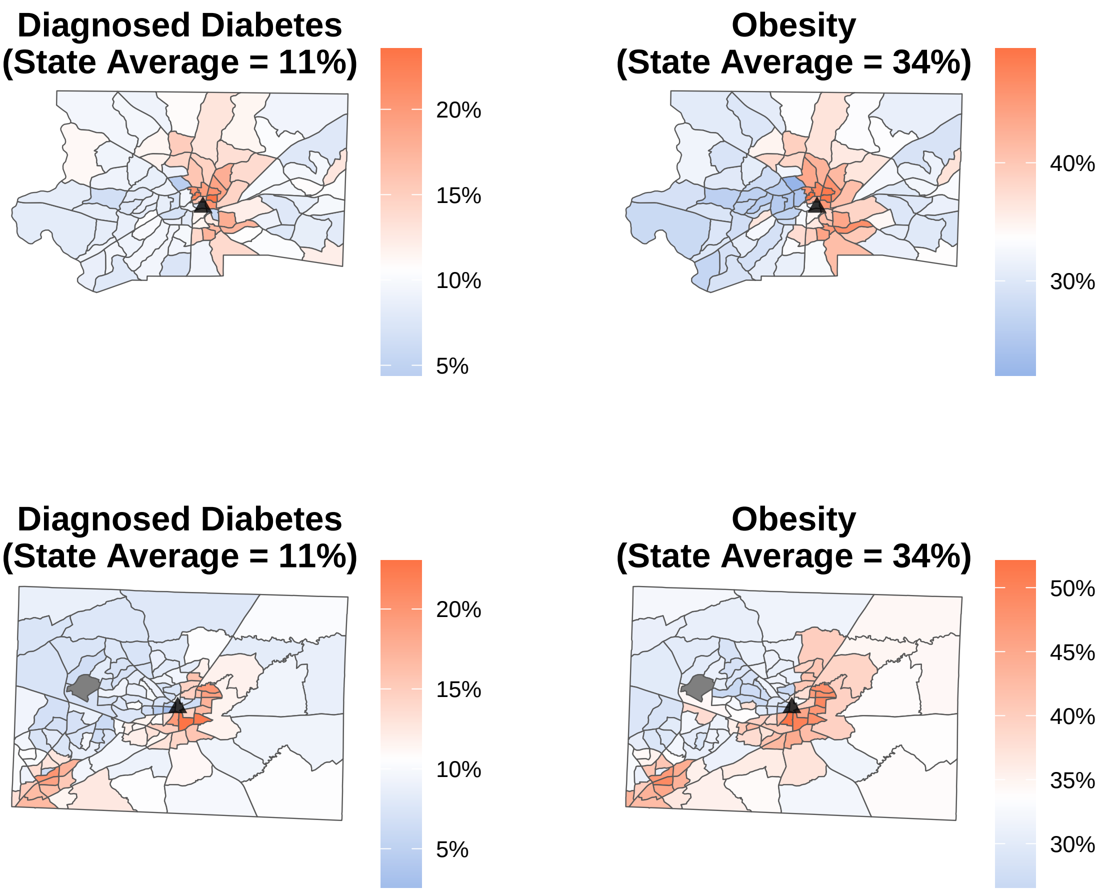

Figure S9: Choropleth maps of the crude prevalence of adverse health outcomes for census tracts in Forsyth County (top row) and Guilford County (bottom row), North Carolina. The triangles denote "downtown" Winston-Salem and Greensboro (represented by their City Halls) in the top and bottom row, respectively. The gradient for each map is centered at the state average (median). Data were taken from the 2022 PLACES dataset (Centers for Disease Control and Prevention, 2022).

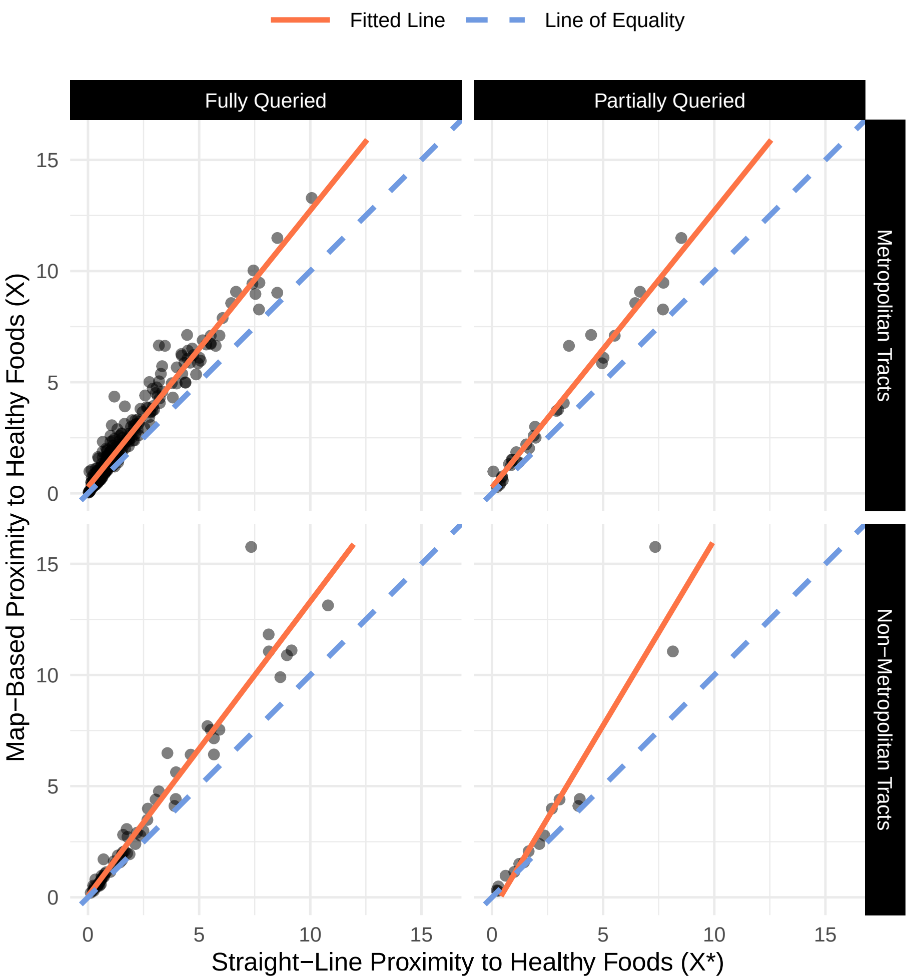

Figure S10: Scatter plot of straight-line versus map-based proximity to healthy food store for neighborhoods (census tracts) in the Piedmont Triad, North Carolina using the fully queried data ($N = 387$) or the partially queried data ($n = 48$). The top row is among only metropolitan census tracts, and the bottom row is only among non-metropolitan census tracts. The solid line follows the fitted least-squares linear regression between X and X^* among those tracts, while the dashed line denotes the hypothetical $X = X^*$ if there had been no errors in X^* (i.e., the line of equality).

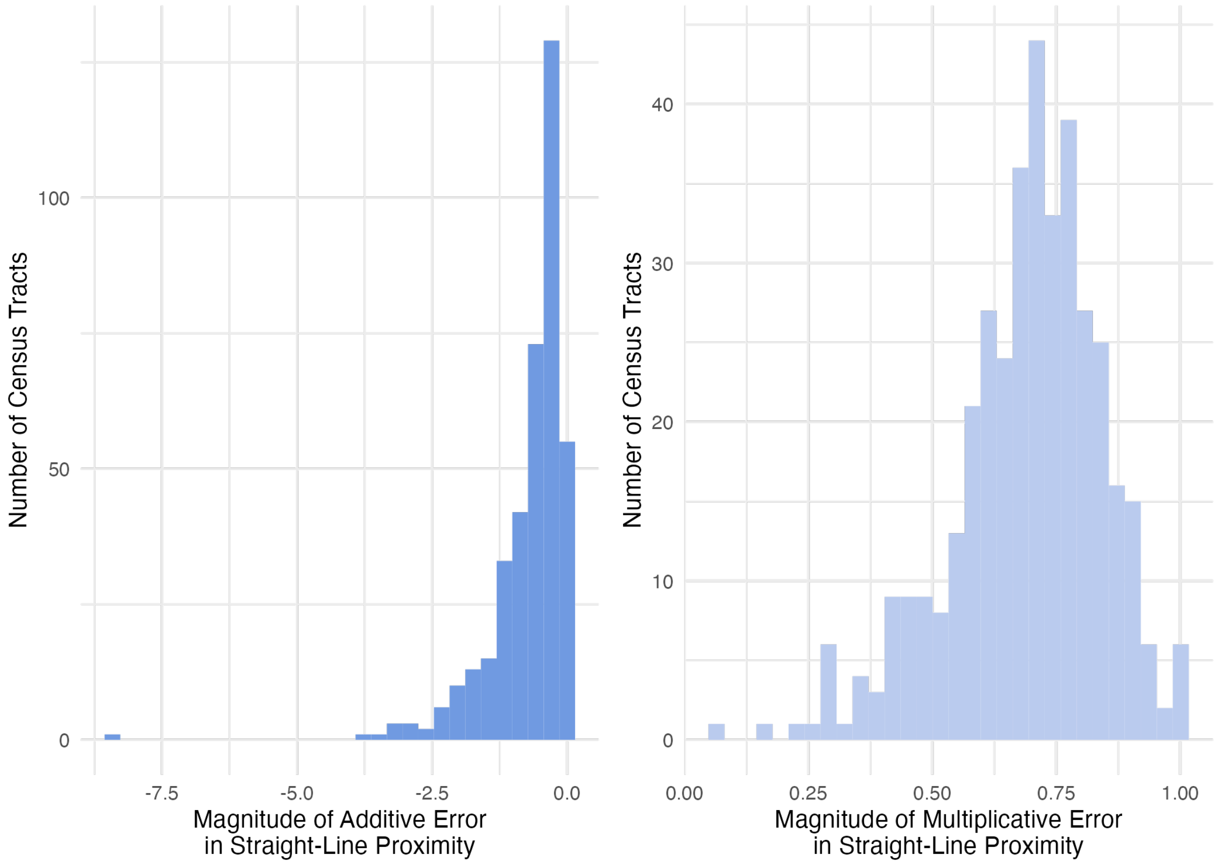

Figure S11: Histogram of additive errors (U) and multiplicative errors (W) in straight-line proximity to healthy foods (X^*) from the fully queried data ($N = 387$) for the Piedmont Triad, North Carolina.

S.2 Simulations with Spatial Autocorrelation

In the simulation studies in the main text (Section 3), all neighborhoods were generated independently. Here, we explore the gold standard, naive, complete case, and imputation approaches to estimate the mixed-effects model in Equation (2) under different amounts of variability in the spatial random effect.

Samples of $N = 387$ or 2168 neighborhoods were considered, where the actual adjacency matrices for the Piedmont Triad and all of North Carolina, respectively, were used. (One census tract outside of the Piedmont Triad that had zero neighbors had to be excluded due to matrix singularity issues, so we reduced $N = 2169$ to 2168 for just these simulations.) Error-free and error-prone proximity, X and X^*, respectively, along with the model offset, Pop, were simulated following Section 3.1, and a proportion of $q = 0.1$ neighborhoods were queried to have non-missing X.

As in Section 4.6, the random intercepts follow a conditional autoregressive (CAR) model, which implies that the vector $\boldsymbol{r} = (r_1, \dots, r_N)^\top$ has a multivariate normal distribution with mean vector $\mathbf{0}$. The $N \times N$ covariance matrix for the CAR model is $\boldsymbol{\Sigma} = \tau^2(\boldsymbol{D}_A - \rho\boldsymbol{A})^{-1}$, where τ^2 relates to the variance, ρ is a spatial autocorrelation parameter, $\boldsymbol{A}$ is the $N \times N$ adjacency matrix, and $\boldsymbol{D}_A$ is the $N \times N$ matrix with the row sums of $\boldsymbol{A}$ (i.e., the number of bordering neighborhoods for each) along the diagonal.

Using the *MASS* package in R (Venables and Ripley, 2002), the vector $\boldsymbol{r}$ was simulated from a multivariate normal distribution with mean $\mathbf{0}$ and covariance $\boldsymbol{\Sigma}$, as described above, with $\rho = 0.9$ and $\tau^2 \in \{0, 0.01, 0.07, 0.14\}$. With $\tau^2 = 0.07$ observed for diabetes prevalence in the Piedmont Triad data, less and more variability for the random effects was considered. Finally, the number of cases Y was generated from a Poisson distribution with mean $=$ $Pop\,\{\exp(-2.2 + 0.01X + r)\}$ (for $r \in \boldsymbol{r}$).

Then, the model in Equation (2) was of interest, which captured the association between the neighborhood-level prevalence of the outcome (Y/Pop) and map-based proximity to healthy food stores (X) using mixed-effects Poisson regression. The `fitme` function from the *spaMM* package in R (Rousset and Ferdy, 2014) was used with the gold standard,

naive, complete case, and imputation analysis approaches. For imputation, each unqueried neighborhood's missing map-based proximity X was imputed $B = 20$ times.

Simulation results under different amounts of variability in the spatial random effect are summarized in Table S4. In all settings considered, the imputation estimator exhibited low bias ($< 2\%$), coverage probabilities close to the nominal 95%, and much higher relative efficiency to the gold standard ($0.64 - 0.67$) than the complete case analysis ($0.08 - 0.10$).

Table S4: Simulation results for the mixed-effects model under additive errors in straight-line proximity to healthy foods, with fixed $\mu_U = -0.7$ and $\sigma_U = 0.8$, and different amounts of variability in the spatial random effect, as controlled by τ^2.

		**Gold Standard**		**Naive**		**Complete Case**			**Imputation**				
N	τ^2	**Bias**	**ESE**	**Bias**	**ESE**	**Bias**	**ESE**	**RE**	**Bias**	**ESE**	**ASE**	**CP**	**RE**
387	0.00	−0.003	0.001	0.043	0.001	−0.003	0.003	0.087	−0.017	0.001	0.001	0.964	0.636
	0.01	0.001	0.001	0.047	0.001	0.007	0.005	0.085	−0.005	0.002	0.002	0.963	0.652
	0.07	0.006	0.003	0.053	0.003	0.065	0.009	0.078	0.007	0.003	0.004	0.971	0.696
	0.14	−0.012	0.004	0.038	0.004	0.019	0.013	0.085	−0.008	0.004	0.005	0.970	0.659
2168	0.00	0.002	0.000	0.048	0.000	−0.007	0.001	0.097	−0.005	0.000	0.001	0.971	0.662
	0.01	0.000	0.001	0.046	0.001	−0.003	0.002	0.103	−0.003	0.001	0.001	0.958	0.672
	0.07	0.002	0.001	0.048	0.001	−0.003	0.004	0.090	0.002	0.001	0.002	0.974	0.667
	0.14	−0.007	0.002	0.041	0.002	−0.024	0.005	0.089	−0.007	0.002	0.002	0.972	0.664

Note: **Bias** and **ESE** are, respectively, the empirical relative bias and standard error of the log prevalence ratio estimator $\hat{\beta}_1$; **ASE** is the average of the standard error estimator $\widehat{\text{SE}}(\hat{\beta}_1)$; **CP** is the empirical coverage probability of the 95% confidence interval for the log prevalence ratio β_1; **RE** is the empirical relative efficiency to the Gold Standard. All entries are based on 1000 replicates.

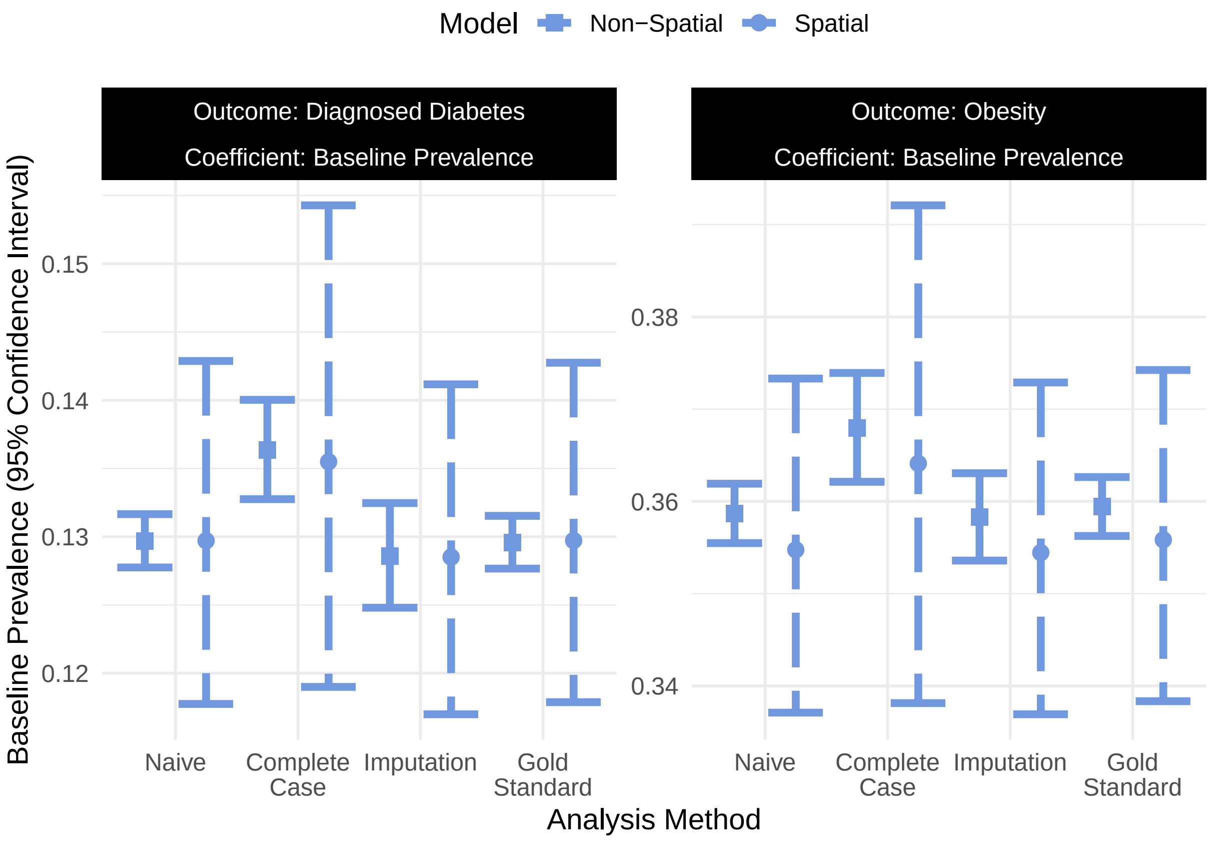

Figure S12: Estimated baseline prevalence (with 95% confidence intervals) for the two health outcomes (diabetes and obesity) in the Piedmont Triad, North Carolina using four different analysis methods. These "baseline" estimates refer to non-metropolitan census tracts with 0 miles proximity to healthy foods. Within each health outcome and method, estimates on the right with the dashed error bars came from the mixed-effects model allowing for spatial autocorrelation between bordering neighborhoods; estimates on the left with the solid error bars came from the non-spatial model assuming independence between neighborhoods.

References

Centers for Disease Control and Prevention (2022). PLACES. https://www.cdc.gov/places. [Online; accessed 20-April-2023].

D'Agostino McGowan, L., S. C. Lotspeich, and S. A. Hepler (2024). The 'Why' behind including 'Y' in your imputation model. *Stat Methods Med Res 33*(6), 996–1020.

Moons, K. G. M., R. A. R. T. Donders, T. Stijnen, and F. E. Harrell (2006). Using the outcome for imputation of missing predictor values was preferred. *Clin Epidemiol 59*(10), 1092–1101.

Rousset, F. and J.-B. Ferdy (2014). Testing environmental and genetic effects in the presence of spatial autocorrelation. *Ecography 37*(8), 781–790.

United States Department of Agriculture (2022). Historical SNAP retailer locator data. https://www.fns.usda.gov/snap/retailer/historicaldata. [Online; accessed 21-July-2023].

Venables, W. N. and B. D. Ripley (2002). *Modern Applied Statistics with S* (Fourth ed.). New York: Springer. ISBN 0-387-95457-0.
