# Supplementary figures and images for: Combining Straight‐Line and Map‐Based Distances to Investigate the Connection Between Proximity to Healthy Foods and Disease
Source: Stat Med. 2025 Apr 14;44(7):e70054. doi: 10.1002/sim.70054 (PMC11995689; doi:10.1002/sim.70054)

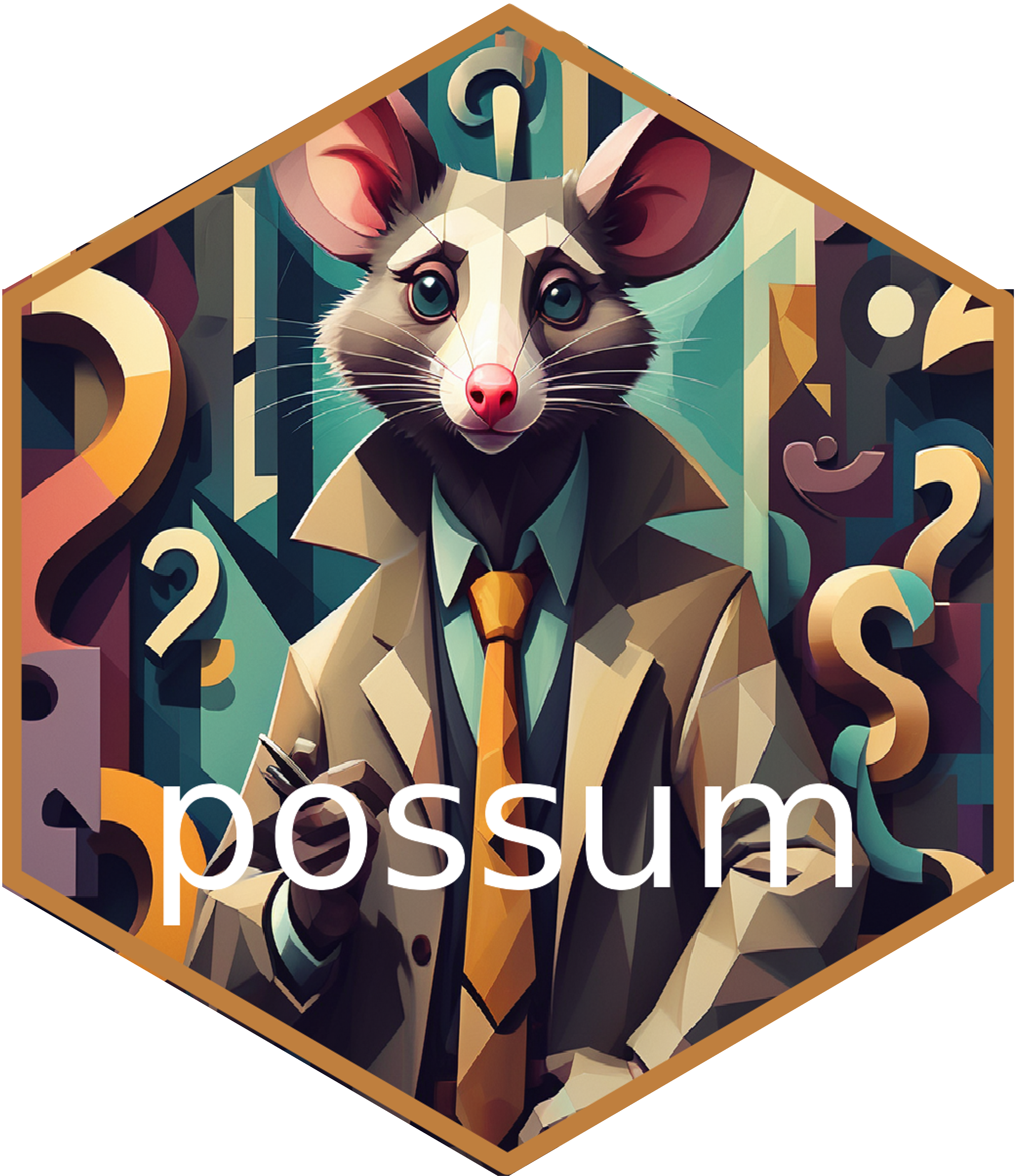

Supplement: Supplementary file 2 — Data S2. Supporting Information. [file SIM-44-0-s003.gz › rpackage/hex.png]
