# Supplementary material for: Combining Straight‐Line and Map‐Based Distances to Investigate the Connection Between Proximity to Healthy Foods and Disease
Source: Stat Med. 2025 Apr 14;44(7):e70054. doi: 10.1002/sim.70054 (PMC11995689; doi:10.1002/sim.70054)
Supplement: Supplementary file 3 — Data S3. Supporting Information. [file SIM-44-0-s001.zip › Data Files/README.pdf]

Combining straight-line and map-based distances to investigate the connection between proximity to healthy foods and disease

This repository contains R code and simulation data to reproduce results from the manuscript by Lotspeich, Mullan, D'Agostino McGowan, and Hepler.

These simulations rely on the `possum` package, which implements the multiple imputation approach for covariate measurement error in Poisson regression from the paper. The package can be found on GitHub, at https://github.com/sarahlotspeich/possum, and installed in R as follows:

```
# Run once
# install.packages("devtools")
devtools::install_github("sarahlotspeich/possum", ref = "main")
```

Primary Data Source (Analysis)

1. Merged Analytical Dataset created using the Healthy Food Store Locations, Disease Prevalence by Neighborhood (Census Tract), Neighborhood (Census Tract) Population Centers, and Rural-Urban Commuting Area (RUCA) Codes.

- Script (Calculate Proximity): *Data1-Merged-Analytical-Dataset/Script1-Calculate-Proximity*
  - *Note:* A small number of census tract population centers could not be geocoded based on the original address. They were manually reviewed and assigned the nearest address that *would* geocode, and then their proximity to healthy foods was recalculated.
  - See the raw data (*Data1-Merged-Analytical-Dataset/raw_proximity_healthy_foods.csv*) and script (*Data1-Merged-Analytical-Dataset/Script2-Review-Proximity*) for more details.
- Data (Proximity): *Data1-Merged-Analytical-Dataset/review_proximity_healthy_foods.csv*
- Data (Food Access + Health + RUCA) *Data1-Merged-Analytical-Dataset/analysis_data.csv*

2. Healthy Foods Store Locations taken from the United States Department of Agriculture's (UDSA's) Historical Supplemental Nutrition Assistance Program (SNAP) Retail Locator Data (2022 Release).

- Download Data: https://www.fns.usda.gov/snap/retailer-locator
- Script (Filter Data): *Data2-Healthy-Food-Store-Locations/healthy_foods_stores_2022.R*
- Data (Food Stores): *Data2-Healthy-Food-Store-Locations/healthy_foods_stores_2022.csv*

3. Disease Prevalence by Neighborhood (Census Tract) taken from the United States Centers for Disease Control and Prevention (CDC's) PLACES Dataset (2022 Release).

- Download Data: https://data.cdc.gov/500-Cities-Places/PLACES-Census-Tract-Data-GIS-Friendly-Format-2022-/shc3-fzig/data
- Script (Filter Data): *Data3-Disease-Prevalence/disease_prevalences_2022.R*
- Data (Disease Prevalence): *Data3-Disease-Prevalence/disease_prevalences_2022.csv*

4. Neighborhood (Census Tract) Population Centers taken from the United States Census Bureau (2010 Release).

- Download Data (Population Centers): https://www2.census.gov/geo/docs/reference/cenpop2010/tract/CenPop2010_M

5. Rural-Urban Commuting Areas (RUCA) taken from the United States Department of Agriculture (2010 Release).

- Download Data (RUCA): https://www.ers.usda.gov/data-products/rural-urban-commuting-area-codes/documentation/

Secondary Data Sources (Descriptive)

6. American Community Survey (ACS) taken from the United States Census Bureau (2015 Release). Data were extracted using the `tidycensus` package.

- Script (Build Data): *Data6-American-Community-Survey/piedmont_triad_acs_data.R*
- Data (Piedmont Triad ACS): *Data6-American-Community-Survey/piedmont_triad_acs_data.csv*

Tables

Table 1. Simulation results under increasing severity of errors in straight-line proximity to healthy foods, as controlled by the error standard deviation σ_U.

- Script: *Simulations/Table1-Sims-Vary-sigmaU.R*

Table 2. Simulation results under increasing proportion of neighborhoods queried to obtain map-based proximity to healthy foods, as controlled by p_V.

- Script: *Simulations/Table2-Sims-Vary-q*

Table 3. Simulation results under higher disease prevalence and prevalence ratios for map-based proximity to healthy foods, as controlled by the coefficients β_0 and β_1, respectively.

- Script: *Simulations/Table3-Sims-Vary-Prev-PR.R*

Table 4. Simulation results under varied multiplicative errors in straight-line proximity to healthy foods, as controlled by the max of the error distribution τ_W.

- Script: *Simulations/Table4-Sims-Mult-Error-Vary-sigmaW.R*

Table S1. Simulation results under varied additive errors in straight-line proximity to healthy foods, as controlled by the mean μ_U of the errors U. The standard deviation $\sigma_U = 0.8$ of the errors was fixed.

- Script: *Simulations/TableS1-Sims-Vary-muU.R*

Table S2. Simulation results under varied multiplicative errors in straight-line proximity to healthy foods, as controlled by the mean μ_W of the errors W. The standard deviation $\sigma_W = 0.15$ of the errors was fixed.

- Script: *Simulations/TableS2-Sims-Mult-Error-Vary-muW.R*

Table S3. Descriptive statistics of the $N = 387$ census tracts in the Piedmont Triad, North Carolina.

- Script (Make Table): *Analysis/TableS3-Piedmont-Descriptive.R*
- Data (Food Access + Health + RUCA) *Data1-Merged-Analytical-Dataset/analysis_data.csv*

Table S4. Simulation results for the mixed-effects model under additive errors in straight-line proximity to healthy foods, with fixed $\mu_U = -0.7$ and $\sigma_U = 0.8$, and different amounts of variability in the spatial random effect, as controlled by τ^2.

- Script: *Simulations/TableS4-Sims-Spatial.R*

Figures

Figure 1. Choropleth map of food access, as measured by proximity to healthy foods for each neighborhood (census tract) in the Piedmont Triad, North Carolina, according to the data used for the naive, gold standard, imputation, and complete-case analyses.

- Data (Food Access + Health + RUCA) *Data1-Merged-Analytical-Dataset/analysis_data.csv*
- Script (Make Figure): *Figures/Figure1-Map-Piedmont-Proximity.R*

Figure 2. Choropleth maps of the crude prevalence of diagnosed diabetes and obesity for census tracts in the Piedmont Triad, North Carolina.

- Data (Disease Prevalence): *Data3-Disease-Prevalence/disease_prevalences_2022.csv*
- Script (Make Figure): *Figures/Figure2-Map-Piedmont-Health*

Figure 3. Scatter plot of straight-line versus map-based proximity to healthy foods store for neighborhoods in the Piedmont Triad, North Carolina using the fully-queried data ($N = 387$).

- Data (Food Access + Health + RUCA) *Data1-Merged-Analytical-Dataset/analysis_data.csv*
- Script (Make Figure): *Figures/Figure3-Scatterplot-Piedmont-Proximity.R*

Figure 4. Estimated prevalence ratios (with 95% confidence intervals) for proximity to healthy foods, metropolitan status, and their interaction with the two health outcomes in the Piedmont Triad, North Carolina using four different analysis methods. Within each health outcome and method, estimates on the right with the dashed error bars came from the mixed effects model allowing for spatial autocorrelation between neighboring census tracts; estimates on the left with the solid error bars came from the non-spatial model assuming independence between tracts.

- Data (Food Access + Health + RUCA) *Data1-Merged-Analytical-Dataset/analysis_data.csv*
- Script (Fit Models): *Analysis/Fit-All-Models.R*
- Script (Make Figure): *Figures/Figure4-ForestPlot-Piedmont-PRs.R*

Figure S1. Straight-line and map-based distances from Reynolda House (square symbol) to a nearby Food Lion grocery store (triangle symbol) in Winston-Salem, North Carolina.

- Script (Make Figure): *Figures/FigureS1-Map-Comparing-Distances.R*

Figure S2. Line graph of the cumulative computing time (in seconds) for the map-based versus straight-line distance calculations in the Piedmont Triad data.

- Data (Food Access + Health + RUCA) *Data1-Merged-Analytical-Dataset/analysis_data.csv*
- Script (Make Figure): *Figures/FigureS2-Line-Computing-Time.R*

Figure S3. Estimated prevalence ratios for map-based food access X on health using multiple imputation. The five possible ways to include the analysis model outcome Y (with or without the model offset *Pop*) in the imputation model for X were considered.

- Script (Run Simulations): *Simulations/FigureS3-Include-Outcome-Imputation.R*
- Script (Make Figure): *Figures/FigureS3-Boxplot-Include-Outcome-Imputation.R*

Figure S4. Map of the 100 North Carolina counties, colored by whether they belong to the Piedmont Triad ($n = 12$), border the Piedmont Triad ($n = 12$), or fall into the rest of the state ($n = 76$).

- Script (Make Figure): *Figures/FigureS4-NC-County-Map.R*

Figure S5. Choropleth maps of socioeconomic factors across the census tracts of the Piedmont Triad, North Carolina. Data were taken from the 2015 American Community Survey. The gradient for each map is centered at the state median. Two census tracts were missing median family income. All other maps are based on $N = 387$ census tracts. The one census tract with zero population was excluded.

- Data (RUCA): https://www.ers.usda.gov/data-products/rural-urban-commuting-area-codes/documentation/
- Data (Piedmont Triad ACS): *Data6-American-Community-Survey/piedmont_triad_acs_data.csv*
- Script (Make Figure): *Figures/FigureS5-Map-Piedmont-ACS.R*

Figure S6. Choropleth maps of percents of census tract population by self-reported race in the Piedmont Triad, North Carolina. Data were taken from the 2015 American Community Survey. The gradient for each map is centered at the state median. All maps are based on $N = 387$ census tracts. The one census tract with zero population was excluded.

- Data (Piedmont Triad ACS): *Data6-American-Community-Survey/piedmont_triad_acs_data.csv*
- Script (Make Figure): *Figures/FigureS6-Map-Piedmont-Race.R*

Figure S7. Map of $M = 701$ authorized SNAP retailers in the Piedmont Triad, North Carolina, broken down by store type. Data were taken from the 2022 Historical SNAP Retail Locator Data.

- Data (Food Stores): *Data2-Healthy-Food-Store-Locations/healthy_foods_stores_2022.csv*
- Script (Make Figure): *Figures/FigureS7-Map-Healthy-Food-Stores.R*

Figure S8. Map of census tracts in the Piedmont Triad, North Carolina, colored according to whether it was treated as queried in the partially queried analysis. For the partially queried analysis, $n = 48$ tracts were chosen to be queried via county-stratified random sampling. The thicker boundaries denote outline the 12 counties.

- Data (Food Access + Health + RUCA) *Data1-Merged-Analytical-Dataset/analysis_data.csv*
- Script (Make Figure): *Figures/FigureS8-Map-Piedmont-Queried-Tracts.R*

Figure S9. Choropleth maps of the crude prevalence of adverse health outcomes for census tracts in Forsyth County (top row) and Guilford County (bottom row), North Carolina. The triangles denote "downtown" Winston-Salem and Greensboro (represented by their City Halls) in the top and bottom row, respectively. The gradient for each map is centered at the state median. Data were taken from the 2022 PLACES dataset.

- Data (Disease Prevalence): *Data3-Disease-Prevalence/disease_prevalences_2022.csv*
- Script (Make Figure): *Figures/FigureS9-Map-Forsyth-Guilford-Health.R*

Figure S10. Scatter plot of straight-line versus map-based proximity to healthy food store for neighborhoods (census tracts) in the Piedmont Triad, North Carolina using the fully queried data ($N = 387$) or the partially queried data ($n = 48$). The top row is among only metropolitan census tracts, and the bottom row is only among non-metropolitan census tracts. The solid line follows the fitted least-squares linear regression fit between X and X^* among those tracts, while the dashed line denotes the hypothetical $X = X^*$ if there had been no errors in X^*.

- Data (Food Access + Health + RUCA) *Data1-Merged-Analytical-Dataset/analysis_data.csv*
- Script (Make Figure): *Figures/FigureS10-Scatterplot-Piedmont-Proximity-By-Metro.R*

Figure S11. Histogram of additive errors (U) and multiplicative errors (W) in straight-line proximity to healthy foods (X^*) from the fully queried data ($N = 387$) for the Piedmont Triad, North Carolina.

- Data (Food Access + Health + RUCA) *Data1-Merged-Analytical-Dataset/analysis_data.csv*
- Script (Make Figure): *Figures/FigureS11-Histogram-Piedmont-Errors.R*

Figure S12. Estimated baseline prevalence (with 95% confidence intervals) for health outcomes in the Piedmont Triad, North Carolina using different analysis methods. Within each health outcome and method, estimates on the right came from the mixed effects model allowing for spatial autocorrelation between bordering neighborhoods (census tracts); estimates on the left came from the model assuming independence between neighborhoods.

- Data (Food Access + Health + RUCA) *Data1-Merged-Analytical-Dataset/analysis_data.csv*
- Script (Fit Models): *Analysis/Fit-All-Models.R*
- Script (Make Figure): *Figures/FigureS12-ForestPlot-Piedmont-Intercept.R*
